# 3’UTR Polymorphism in ACSL1 Gene Correlates with Expression Levels and Poor Clinical Outcome in Colon Cancer Patients

**DOI:** 10.1371/journal.pone.0168423

**Published:** 2016-12-19

**Authors:** Teodoro Vargas, Juan Moreno-Rubio, Jesús Herranz, Paloma Cejas, Susana Molina, Marta Mendiola, Emilio Burgos, Ana B. Custodio, María De Miguel, Roberto Martín-Hernández, Guillermo Reglero, Jaime Feliu, Ana Ramírez de Molina

**Affiliations:** 1 Molecular Oncology, IMDEA-Food Institute, CEI UAM+CSIC, Madrid, Spain; 2 Precision Oncology Laboratory (POL), Infanta Sofía University Hospital, San Sebastián de los Reyes, Madrid, Spain; 3 Translational Oncology Laboratory, La Paz University Hospital (IdiPAZ), Madrid, Spain; 4 Biostatistics and Bioinformatics Unit, IMDEA-Food Institute, CEI UAM+CSIC, Madrid, Spain; 5 Department of Medical Oncology, Dana-Farber Cancer Institute, Boston, MA, United States of America; 6 Pathology Department, La Paz University Hospital (IdiPAZ), Madrid, Spain; 7 Medical Oncology, La Paz University Hospital (IdiPAZ), Madrid, Spain; Sapporo Ika Daigaku, JAPAN

## Abstract

Strong evidence suggests that lipid metabolism (LM) has an essential role in tumor growth to support special energetic and structural requirements of tumor cells. Recently, overexpression of LM-related genes, apolipoproteins related to metabolic syndrome, and ACSL/SCD network involved in fatty acid activation have been proposed as prognostic markers of colon cancer (CC). Furthermore, activation of this latter lipid network has been recently demonstrated to confer invasive and stem cell properties to tumor cells promoting tumor aggressiveness and patient relapse. With the aim of elucidating whether any genetic variation within these genes could influence basal expression levels and consequent susceptibility to relapse, we genotype, in 284 CC patients, 57 polymorphisms located in the 7 genes of these lipid networks previously associated with worse clinical outcome of CC patients (*ABCA1*, *ACSL1*, *AGPAT1*, *APOA2*, *APOC1*, *APOC2* and *SCD*), some of them related to CC aggressiveness. After adjusting with clinical confounding factors and multiple comparisons, an association between genotype and disease-free survival (DFS) was shown for rs8086 in 3’-UTR of *ACSL1* gene (HR 3.08; 95% CI 1.69–5.63; adjusted p = 0.046). Furthermore, the risk T/T genotype had significantly higher *ACSL1* gene expression levels than patients carrying C/T or C/C genotype (means = 5.34; 3.73; 2.37 respectively; p-value (ANOVA) = 0.019), suggesting a functional role of this variant. Thus, we have identified a “risk genotype” of *ACSL1* gene that confers constitutive high levels of the enzyme, which is involved in the activation of fatty acids through conversion to acyl-CoA and has been recently related to increased invasiveness of tumor cells. These results suggest that rs8086 of *ACSL1* could be a promising prognostic marker in CC patients, reinforcing the relevance of LM in the progression of CC.

## Introduction

Colorectal cancer (CRC [MIM: 114500]) is one of the most common neoplasms worldwide, and represents the third most frequent cancer in men (746 000 cases, 10%) and the second in women (614 000 cases, 9.2%). In Europe CRC represents the second cause of cancer deaths, estimating 113 000 deaths in men (11.6%) and 101 000 in women (13%), and only behind lung cancer (26.1%) and breast cancer (16.8%) respectively [1].

The pathogenesis of CRC is extremely complex and implicates sequential genetic and epigenetic mechanisms, which in many cases remain to be elucidated. Lifestyle factors, nutrition, environment, as well as genetic events have been associated with the causality of CRC and survival of patients after diagnosis of CRC [2]. In this sense, obesity has been linked to higher risk of developing CRC [3–5] and several studies have shown an increased risk of about 1.5 to 3 times, and found a 3% increase in the risk of CRC per 1 unit increase in the body mass index [5, 6]. Epidemiological studies have reported that in Europe around 11% of CRC cases have been attributed to overweight and obesity. Thus, obesity is associated with worse cancer outcome, including recurrence of the primary cancer or mortality [7]. Indeed, obesity has been significantly associated with CC recurrence and death in patients with curatively resected stage II and III cancers treated with adjuvant chemotherapy, and this association was more evident in patients who had severe obesity compared with normal-weight patients. Furthermore, obesity compared with normal-body weight was significantly associated with an increased number of lymph node metastases, an established worse prognostic factor in CC [8, 9].

In this sense, we have recently reported that several genes traditionally linked to metabolic syndrome and obesity such as apolipoproteins A2, C1 or C2 play also a relevant role in CC progression, reporting for the first time a genetic link among these diseases. Furthermore, we have found that LM activation specifically through Acyl-CoA synthetase/ Stearoyl-CoA desaturase ACSL/SCD network, is associated to CC patient relapse due to phenotypic plasticity associated to this metabolic reprogramming [10–12].

Surgical resection is the only curative treatment modality for localized CC (stage I-III), and adjuvant chemotherapy is recommended for high-risk stage II and all stage III tumors [13]. While adjuvant chemotherapy is standard for stage III CC patients because reduces risk of recurrence and prolongs DFS [13] its use in stage II CC patients is controversial [14]. However, even with adjuvant chemotherapy, 20%-30% of high-risk stage II and 30%-40% of stage III patients relapse within 5 years [13]. In current clinical practice, the majority of these intermediate stage CC patients receive adjuvant treatment unnecessarily, either because they were cured by surgery alone or because they will relapse despite adjuvant treatment [14]. Consequently, it is essential to identify markers that might classify patients who will benefit from adjuvant therapy, and avoid the toxic and unnecessary chemotherapy in patients who will relapse despite adjuvant treatment. In this regard, in our recent analysis of LM alterations associated to CC progression, we identify a gene expression signature of 4 LM-related genes (ColoLipidGene) with a strongly marked role in stage II CC patients prognosis [11]. We observed that the combined activation of lipid transport through *ABCA1* (ATP-Binding Cassette Subfamily-A Member 1 (*ABCA1* [MIM: +600046]), lipid activation through *ACSL1* and *AGPAT1* (Acyl-CoA Synthetase Long Chain Family, Member 1 (*ACSL1* [MIM: *152425]), 1-Acylglycerol-3-Phosphate O-Acyltransferase 1 (*AGPAT1* [MIM: *603099]) and lipid-related toxicity drainage through *SCD* (Stearoyl-Coa Desaturase (*SCD* [MIM: *604031]) might confer an energetic advantage to the tumoral cell resulting in the promotion of tumor progression and relapse [10–12].

Proceeding with this line of research, and with the aim of identifying whether any genetic alteration might be related to overexpression of these enzymes and therefore constitute a biomarker of LM-related alterations, we analyzed in stage II and III CC patients the main polymorphisms within these genes previously identified as potential promoters of the energetic advantage associated with worse clinical outcome of CC patients [11, 12].

## Materials and Methods

### Subjects

This retrospective study consisted of a cohort of 308 stage II and III CC patients who had undergone surgery between 2000 and 2008 in La Paz University Hospital, from which 8 were eliminated due to low quantity of the tumor sample. CC patients were clinically diagnosed based on histopathological criteria by AJCC/UICC and were classified following the clinical risk criteria of the American Society of Clinical Oncology (ASCO), and were randomly selected for this study. Eligibility required histologically confirmed Stage II or III AJCC/UICC primary colorectal cancer, long-term follow-up among survivors (>3 years) and age ≥ 18, completely resected colon adenocarcinoma located at ≥15 cm of the anal verge as determined by endoscopy or above the peritoneal reflection in the surgical resection. Additional eligibility criteria included good quality of RNA sample. Patients who died within 30 days after surgery, patients with incompletely excised tumor, mixed histological features or other cancers in the previous 5 years were ineligible for this study. Tumor samples were obtained with the approval of The Ethics Committee for Clinical Research (CEIC) of La Paz University Hospital (Madrid) (approval reference: HULP-PI-1452) and were stored embedded in paraffin. All subjects gave their written informed consent to participate in the study and were previously included in gene expression association studies [11, 12].

Clinical data for the CC cases were retrieved from the registry managed by oncologists of La Paz University Hospital (Table 1).

**Table 1:** Clinical characteristics of stage II and III CC patients (n = 284).

Variable	CC Stage II (n = 157)	CC Stage III (n = 127)
	Number (%)	Number (%)
**Sex**		
Male	92 (58.6)	66 (51.97)
Female	65 (41.4)	61 (48.03)
**Age (years)**		
Mean (SD)	67.55 (12.2)	64.34 (11.73)
Range	23–92	23–85
**pT category**		
1–3	107 (68.15)	98 (77.17)
4	50 (31.85)	29 (22.83)
**pN category**		
0	157 (100)	0
1	0	87 (68.5)
2	0	40 (31.5)
**Stage**		
II	157 (100)	0
III	0	127 (100)
**Lymph Nodes Resected**		
≤12	80 (52.3)	39 (30.72)
>12	73 (47.68)	88 (69.3)
Unknown	4	0
**Tumour site**		
Cecum and Ileocecal Valve	11 (7.05)	19 (14.96)
Acending colon and Hepatic flexure	46 (29.49)	19 (14.96)
Transverse colon	10 (6.41)	12 (9.45)
Splenic flexure and Descending colon	19 (12.18)	17 (13.39)
Sigmoid colon and rectosigmoid junction	70 (44.87)	60 (47.24)
Unknown	1	0
**Differentiation Grades**		
Well	12 (7.64)	8 (6.3)
Moderately	130 (82.8)	99 (77.95)
Poor	15 (9.55)	20 (15.75)
**Vascular invasion**		
Yes	42 (27.1)	56 (44.09)
No	113 (72.9)	71 (55.91)
Unknown	2	0
**Neuronal invasion**		
Yes	28 (18.06)	50 (39.37)
No	127 (81.94)	77 (60.63)
Unknown	2	0
**Peritoneal perforation or obstruction**		
Yes	44 (28.03)	37 (29.13)
No	113 (71.97)	90 (70.87)
**Adjuvant chemotherapy**		
5-FU/LV or XELOX or FOLFOX	98 (62.42)	127 (100)
No treatment	59 (37.58)	0
**Disease-free survival**		
Relapses	29 (18.47)	46 (36.22)
**Overall survival**		
Exitus	17 (10.9)	24 (18.9)

SD, standard deviation; 5-FU/LV, 5-Fluorouracil-Leucovorin; XELOX, Capecitabine plus Oxaliplatin; FOLFOX, Oxaliplatin plus 5-FU/LV.

Additionally, we included in this study 40 samples of healthy human colon tissues that were obtained from apparently healthy tissues adjacent to tumors from the patients.

### Candidate genes and SNP selection

Genes were selected according to their key role in the LM and their association in CC prognosis on the basis of previously published gene expression studies [11, 12]. Genetic variants were chosen from the set of common single-nucleotide polymorphisms (SNPs) genotyped in the Caucasian population sample of the HapMap project (Data Release 28/phaseII+III August10, on National Center for Biotechnology Information B36 assembly, dbSNP build 126). The software Haploview version 4.2 (Broad Institute of MIT and Harvard) was used to evaluated haplotype blocks in each gene, as well as to select haplotype tagging SNPs (htSNPs), capturing the variations of all SNP alleles within the gene region with r2 threshold 0.8. The gene region was defined as an extent of genomic DNA + 5 kb approximately upstream and downstream from the first base of the first known exon to the last base of the last known exon. SNPs were selected with main emphasis on their tagging characteristics and on their location (non-synonymous mutation located in exons or functional SNPs located in putative gene regulatory regions, such as promoter regions, 5’ or 3’UTR). We delimited the selection to markers, which in HapMap had a minor allelic frequency (MAF) of at least 5% (with rare and justified exceptions). For TaqMan® SNP Genotyping Assays, we further selected SNPs with low probability of genotyping failure.

Allele frequency and location for each SNP was based on dbSNP (National Center for Biotechnology Information, National Institutes of Health).

Following these criteria, a total of 57 SNPs in 7 genes were selected (Table 2). Eighteen out of the 57 SNPs selected in this study (31,6%) were functional SNPs (located in 5´ near gene, 5’-UTR or 3’-UTR regions) or were in linkage disequilibrium (LD) with some SNP located in these putative gene regulatory regions and 7 SNPs were Missense Mutation (12,3%).

**Table 2:** Location and SNP type of investigated polymorphisms.

Assay ID	dbSNP	Gene Symbol	NCBI Assembly Location	SNP Type
C__11453334_10	rs5082	(5' near *APOA2* gene)	ch. 1: 161193683	**5' near gene; Intron; Transition Substitution**
C____904974_10	rs439401	(near *APOC1* gene)	ch. 19: 45414451	**Intergenic; Transition Substitution; LD with rs584007 (5' near gene)**
C__11466277_30	rs1064725	*APOC1*	ch. 19: 45422561	**3'-UTR; Transversion Substitution**
C__15880051_10	rs2288911	*APOC2*	ch. 19: 45449284	**5'-UTR; Transversion Substitution**
C___1345738_10	rs3870747	*SCD*	ch. 10: 102113679	**Intron; Transition Substitution; LD with rs11557927 (3' UTR)**
C___1345731_10	rs508384	(near *SCD* gene)	ch. 10: 102124761	**Intron; Transversion Substitution; LD with rs7849 (3' UTR)**
C____623412_10	rs599961	*SCD*	ch. 10: 102117207	**Intron; Transversion Substitution; LD with rs560792 (3' UTR)**
C___8734182_10	rs1502593	*SCD*	ch. 10: 102109202	Intron; Transition Substitution
C__44899326_10	rs2234970	*SCD*	ch. 10: 102116311	**Missense Mutation; Transversion Substitution**
C__31980235_10	rs11190483	*SCD*	ch. 10: 102113649	Intron; Transition Substitution
C___9260122_10	rs522951	*SCD*	ch. 10: 102110901	Intron; Transversion Substitution
C___1345737_10	rs3829160	*SCD*	ch. 10: 102115007	Intron; Transition Substitution
C___8242163_10	rs8086	*ACSL1*	ch. 4: 185677421	**3'-UTR; Transition Substitution**
C__29419656_10	rs4069938	*ACSL1*	ch. 4: 185700776	Intron; Transversion Substitution
C___1170092_10	rs4862417	*ACSL1*	ch. 4: 185690601	**Intron; Transition Substitution; LD with rs2292899 (3' UTR)**
C___1170045_10	rs12503643	*ACSL1*	ch. 4: 185746088	Intron; Transversion Substitution
C__30469648_10	rs6552828	*ACSL1*	ch. 4: 185725416	Intron; Transition Substitution
C___8242164_10	rs1056896	*ACSL1*	ch. 4: 185677363	**3'-UTR; Transition Substitution**
C__11785598_10	rs12644905	(3' near *ACSL1* gene)	ch. 4: 185676683	**3' near gene; Intron; Transition Substitution**
C___1170050_1_	rs2280297	*ACSL1*	ch. 4: 185736113	Intron; Transversion Substitution
C___1170082_10	rs7681334	*ACSL1*	ch. 4: 185710859	Intron; Transition Substitution
C___1170059_10	rs13112568	*ACSL1*	ch. 4: 185730299	Intron; Transition Substitution
C___1170066_10	rs11936062	*ACSL1*	ch. 4: 185721370	Intron; Transversion Substitution
C___1170097_10	rs11727009	*ACSL1*	ch. 4: 185687863	Intragenic; Transition Substitution; Silent Mutation
C__15931315_10	rs2777786	*ABCA1*	ch. 9: 107661561	Intron; Transversion Substitution
C___2741051_1_	rs2230806	*ABCA1*	ch. 9: 107620867	**Missense Mutation; Transition Substitution**
C___2741083_1_	rs2066714	*ABCA1*	ch. 9: 107586753	**Missense Mutation; Transition Substitution; Silent Mutation**
C___2741104_1_	rs2230808	*ABCA1*	ch. 9: 107562804	**Missense Mutation; Transition Substitution**
C____500971_10	rs2472449	*ABCA1*	ch. 9: 107604197	Intron; Transversion Substitution
C___2741081_20	rs2066715	*ABCA1*	ch. 9: 107588033	**Missense Mutation; Transition Substitution**
C__31952217_10	rs4149338	*ABCA1*	ch. 9: 107545903	**3'-UTR; Transition Substitution**
C__16235603_10	rs2472496	(near *ABCA1* gene)	ch. 9: 107695353	Intergenic; Transition Substitution
C__15849583_20	rs2740486	*ABCA1*	ch. 9: 107666513	Intron; Transversion Substitution
C__11720789_10	rs2066718	*ABCA1*	ch. 9: 107589255	**Missense Mutation; Transition Substitution**
C__16235415_10	rs2246293	(5' near *ABCA1* gene)	ch. 9: 107690838	**5' near gene; intron; Transversion Substitution**
C___2741115_10	rs363717	*ABCA1*	ch. 9: 107544700	**3'-UTR; Transition Substitution**
C___1139523_20	rs2472377	*ABCA1*	ch. 9: 107687104	Intron; Transition Substitution
C__29854619_10	rs4149340	*ABCA1*	ch. 9: 107544685	**3'-UTR; Transition Substitution**
C__16025972_10	rs2515617	*ABCA1*	ch. 9: 107680915	Intron; Transition Substitution
C___2741040_10	rs2000069	*ABCA1*	ch. 9: 107635869	Intron; Transition Substitution
C__27093081_10	rs2472458	*ABCA1*	ch. 9: 107588015	**Missense Mutation; Transition Substitution**
C__15889845_10	rs2482432	(3' near *ABCA1* gene)	ch. 9: 107543172	**3' near gene; Intron; Transition Substitution**
C__11266744_20	rs2740479	*ABCA1*	ch. 9: 107563437	Intron; Transition Substitution
C___2741044_10	rs4743764	*ABCA1*	ch. 9: 107629104	Intron; Transition Substitution
C__16025975_10	rs2515614	*ABCA1*	ch. 9: 107684318	Intron; Transversion Substitution
C__11720848_10	rs2043664	(near *ABCA1* gene)	ch. 9: 107694245	Intergenic; Transition Substitution
C___9456257_10	rs1800977	*ABCA1*	ch. 9: 107690450	**5'-UTR; Transition Substitution**
C__11720790_1_	rs2065412	*ABCA1*	ch. 9: 107598740	Intron; Transition Substitution
C__16061836_10	rs2740484	*ABCA1*	ch. 9: 107551180	Intron; Transition Substitution
C___2960434_10	rs3847304	*ABCA1*	ch. 9: 107655848	Intron; Transition Substitution
C___8783836_10	rs3847305	*ABCA1*	ch. 9: 107657253	Intron; Transversion Substitution
C__11720774_10	rs2066720	*ABCA1*	ch. 9: 107554069	Intron; Transition Substitution
C__11266782_10	rs4743763	*ABCA1*	ch. 9: 107593182	Intron; Transversion Substitution
C___2741003_10	rs2575876	*ABCA1*	ch. 9: 107665739	Intron; Transition Substitution
C__27301445_10	rs3130284	*AGPAT1*	Chr.6: 32140487	Intron; Transition Substitution
C__27462316_10	rs3130283	*AGPAT1*	Chr.6: 32138545	Intron; Transversion Substitution
C___8847986_20	rs1061807	*AGPAT1*	Chr.6: 32136838	**3'-UTR; Transition Substitution**

"SNP type" in bold indicates nonsynonymous mutation or functional SNPs (located in putative gene regulatory region).

*APOA2*, apolipoprotein A-II; *APOC1*, apolipoprotein C-I; *APOC2*, apolipoprotein C-II; *SCD*, stearoyl-CoA desaturase (delta-9-desaturase); *ACSL1*, acyl-CoA synthetase long-chain family member 1; *ABCA1*, ATP-binding cassette sub-family A member 1; *AGPAT1*, 1-acylglycerol-3-phosphate O-acyltransferase 1; ch., chromosome; LD, Linkage disequilibrium; UTR, untranslated region.

### Genotyping

Genomic DNA from formalin fixated paraffin embedded (FFPE) tissue of the patients was extracted using standard methods (QIAamp DNA FFPE Tissue Kit, Qiagen, Hilden, Germany). DNA samples were genotyped for 57 selected SNPs located in 7 different genes implicated in LM (Table 2) and whose expression have previously been associated with prognosis in stage II CC patients [11, 12]. SNPs were screened using TaqMan® OpenArray® Genotyping Plates (Applied Biosystems, Carlsbad, CA, USA) in a QuantStudio™ 12K Flex system according to the manufacturer’s instructions. Genotype calling was obtained with the Taqman Genotyper Software v1.3 (Applied Biosystems™).

### Gene expression analysis

Formalin-Fixed, Paraffin-Embedded (FFPE) tumor samples from stage II and III CC patients were deparaffinated using Deparaffinization Solution (Qiagen Gmbh, Hilden, Germany). Afterwards, total RNA was purified from all samples using RNeasy FFPE Kit (Qiagen) following manufacturer's instructions and then was reverse transcribed by High Capacity cDNA Archive Kit (Applied Biosystems, Carlsba, CA, USA) for 2 h at 37°C, as described in detail in previous studies [11, 12].

Gene expression data for the selected candidate genes (calculated with the 2^–ΔCt^ method) were previously analyzed in a HT-7900 Fast Real time PCR System using Taq-Man Low Density Arrays (Applied Biosystems) and the gene expression data were normalized using the geometric mean of the internal control genes *GAPDH* (Glyceraldehyde 3-phosphate dehydrogenase) and *B2M* (Beta-2 microglobulin) using Real time StatMiner software (Integromics® Inc., Madison, WI, USA) as previously described [11, 12].

### Statistical analysis

Genotype data for the investigated 57 SNPs in 7 candidate genes were obtained as described in “genotyping” section. After Data Quality control and Quality Assurance (QC/QA) process we excluded 19 SNPs that met any of following criteria: minor allele frequency (MAF) < 5%, Hardy-Weinberg equilibrium (HWE) P-value < 0.0001 or percentage of missing data > 5%. Therefore, 38 out of the 57 SNPs were selected, categorized by genotype (homozygote minor allele, heterozygote and homozygote major allele) and checked for additive, dominant and recessive model.

Two-tailed Pearson and Fisher exact tests were used to compare genotype distributions or allele frequencies. Bonferroni corrections for multiple comparisons were performed based in the number of selected SNPs after QC/QA process.

In order to assess the prognostic value of polymorphisms, genotypes of each polymorphism were tested for association with DFS using univariate Cox-regression analysis, expressed as the hazard ratio (HR) with 95% confidence intervals (CI). To calculate the effect on survival with adjustment for potential confounding factors, a multivariate Cox-regression analysis was used including only variables that were significant (p<0·05) in the univariate analysis of the clinical data (S1 Table). DFS was defined as the time from surgery until the first documented tumor recurrence or death. Overall survival was defined as the time from surgery until death. The Kaplan–Meier method was used to estimate the survival probabilities, and the log-rank test was used to test differences between subgroups. Haploview 4.2 software [15] was used to estimate the linkage disequilibrium between the different SNPs.

To evaluate the association between *ACSL1* and *SCD* gene expression level and the different genotypes from the diverse models of inheritance for *ACSL1* rs8086 and *SCD* rs522951 SNPs, a non-parametric Kruskal-Wallis (KW) test and Analysis of Variance test (ANOVA) was performed. Expression data of *ACSL1* and *SCD* genes (calculated with the 2^–ΔCt^ method) were previously analyzed and presented in Vargas et al., 2015 [11].

All statistical calculations were carried out using the R statistical software version 2.15 (www.r-project.org). P values <0.05 were considered significant, and all tests were two sided.

## Results

### Analysis of genetic variants within LM-related genes in CC patients

We aim to analyze the impact of 57 SNPs in 7 LM-related genes (whose expression have previously been associated with worse clinical outcome in stage II CC patients [11, 12]) on DFS in 284 CC patients (Table 2).

We found that among the SNPs selected after QC/QA process, only two genetic variants were associated with clinical outcome of the patients, precisely one in Acyl-CoA Synthetase Long Chain Family member 1 (*ACSL1*) and the other in the Stearoyl-Coa Desaturase gene (*SCD*). Thus, T/T genotype for rs8086 (*ACSL1* gene) in the recessive model of inheritance (HR 3.02; 95% CI 1.66–5.49; p = 0.001) and C/C genotype for rs522951 (*SCD* gene) in the dominant model (HR 0.45; 95% CI 0.28–0.71; p = 0.001) were significantly associated with the clinical outcome of stage II and III CC patients (Table 3).

**Table 3:** Multivariate Cox Regression analyses for DFS of different genetic models of inheritance for rs8086 (*ACSL1*) and rs522951 (*SCD*) SNPs in stage II and III CC patients.

		Bonferroni corrections for multiple comparisons^#^			Adjusted for clinical variables*		
SNP reference	Model	HR	p-value	Adjusted p-value	HR	p-value	Adjusted p-value
**rs8086**	Additive	1.67 (1.16–2.39)	0.006	0.236	1.67 (1.16–2.42)	0.007	0.251
	Dominant	1.43 (0.89–2.3)	0.134	1	1.42 (0.87–2.3)	0.151	1
	Recessive	3.02 (1.66–5.49)	0.001	0.053	3.08 (1.69–5.63)	0.001	0.046
**rs522951**	Additive	0.67 (0.47–0.95)	0.024	0.920	0.67 (0.47–0.95)	0.022	0.817
	Dominant	0.45 (0.28–0.71)	0.001	0.042	0.46 (0.29–0.73)	0.002	0.065
	Recessive	0.99 (0.56–1.74)	0.967	1.000	0.91 (0.51–1.61)	0.741	1.000

^#^Adjustment for Bonferroni method was used in the multiple comparisons.

*Multivariate Cox Regression analyses were adjusted for age>70, pT category, vascular invasion, neuronal invasion and peritoneal perforation or obstruction.

**Fig 1:**
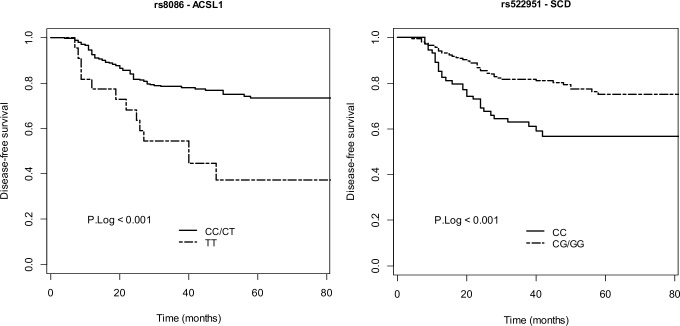
Kaplan-Meier curve of *ACSL1* SNP rs8086 and *SCD* SNP rs522951 on DFS for stage II and III CC patients in a recessive and dominant model of inheritance, respectively. P-value was calculated by Log-rank test.

The Kaplan Meier survival curves and the log-rank test also showed the association between DFS and rs8086 (p<0.001) and rs522951 (p<0.001) (Fig 1).

### *SCD* genetic variant identifies CC patients with high risk of relapse

As mentioned above, C/C genotype for rs522951 (*SCD* gene) in the dominant model was associated with the clinical outcome of CC patients (Fig 1). In the Multivariate Cox Regression analysis, a trend of C/C genotype for rs522951 (p = 0.06; Table 3) with short DFS was observed, indicating that patients carrying the *SCD* rs522951 C/G + G/G genotype had significantly increased DFS compared with patients carrying the C/C genotype. The C/C genotype for rs522951 (24% and 30% for stage II and III respectively, S2 Table) showed more than two-fold higher risk of relapse that patients carrying the C/G + G/G genotype (HR 2.18; 95% CI 1.36–3.5; p = 0.065 (inverse value of HR 0.46; 95% CI 0.29–0.73; p = 0.065)) (Table 3).

Thus, 3-year DFS in patients carrying the “risk genotype” for rs522951 SNP (C/C) was 63% (95%CI: 0.528–0.752) compared with 82% (0.767–0.873) in patients with C/G + G/G genotype (in the dominant model of inheritance).

**Fig 2:**
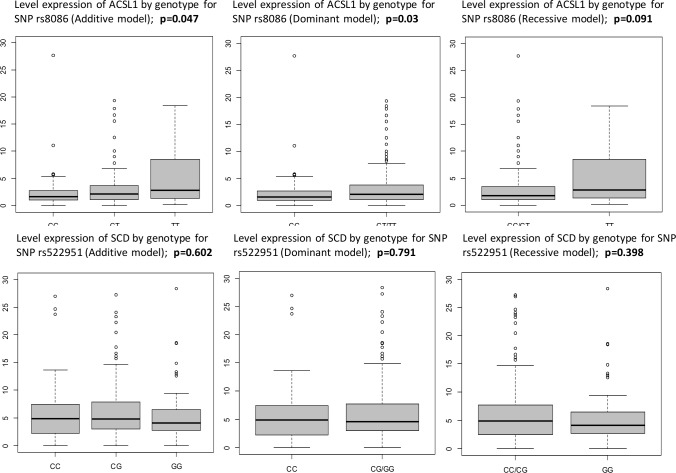
Box plots of the association between gene expression level for *ACSL1* and genotype for rs8086 SNP located on the 3’-UTR region. The box plots show how the *ACSL1* expression values are distributed for each genotype from the Additive, Dominant and Recessive model of inheritance for *ACSL1* rs8086 SNP in stage II and III CC patients. The p-values were calculated using the non-parametric Krustal-Wallis and Mann-Whitney tests, respectively. The line within the box indicate the median of level expression. The gene expression data were normalized using the geometric mean of the internal control genes *GAPDH* and *B2M*.

In addition, since *SCD* over-expression has been associated with tumor progression and early-stage CC patient relapse[11, 16], we investigated whether rs522951 polymorphism correlated with gene expression levels of *SCD* in a subsample of 209 stage II/III CC patients (140 and 69 stage II and III respectively) in which gene expression data was available due to enough amount of sample for DNA and RNA extraction, and gene expression levels had been previously determined [11]. As expected by its intronic localization, results showed no correlation between rs522951 genotype and *SCD* gene expression (Table 4; Fig 2).

**Table 4:** Association between *ACSL1* and *SCD* gene expression level and the different genotypes from the diverse models of inheritance for *ACSL1* rs8086 and *SCD* rs522951 SNPs in stage II and III CC patients.

SNP reference	Model	Genotype	N	Mean	Median	SD	p-value (KW)	p-value (Anova)
**rs8086**	Additive	C/C	90	2.37	1.6	3.22	0.047	0.019
		C/T	103	3.73	2.09	6.9		
		T/T	17	5.34	2.79	5.43		
	Dominant	C/C	90	2.37	1.6	3.22	0.03	0.039
		C/T + T/T	120	3.96	2.15	6.71		
	Recessive	C/C + C/T	193	3.09	1.76	5.53	0.091	0.109
		T/T	17	5.34	2.79	5.43		
**rs522951**	Additive	C/C	55	5.85	4.91	5.63	0.602	0.983
		C/G	111	6.37	4.78	5.55		
		G/G	43	5.81	4.14	5.86		
	Dominant	C/C	55	5.85	4.91	5.63	0.791	0.687
		C/G + G/G	154	6.21	4.65	5.62		
	Recessive	C/C + C/G	166	6.2	4.85	5.56	0.398	0.688
		G/G	43	5.81	4.14	5.86		

The reported p-values correspond to non-parametric Kruskal-Wallis (KW) test and Analysis of Variance test (ANOVA).

Mean and Median of gene expression values for *ACSL1* and *SCD* genes are shown. The quantification of gene expression levels were analyzed in a previously published manuscript (Vargas et al., 2015). The gene expression data were normalized using the geometric mean of the internal control genes *GAPDH* and *B2M*.

N, number of cases with each genotype; SD, standard deviation.

Finally, in order to determine whether this genetic variant was a tumor-specific polymorphism (somatic mutation) or if by contrast was a germline polymorphism of the patient, and therefore putatively traceable in plasma or saliva, we genotyped for rs522951 40 healthy colon samples obtained from tissues adjacent to tumors from these patients. All tumor samples analyzed (100%) showed the same genotype for rs522951 that its respective adjacent CC sample.

### *ACSL1* rs8086 genetic variant is associated with worse clinical outcome of CC patients

On the other hand, T/T genotype for rs8086 (*ACSL1* gene) in the recessive model of inheritance was also found associated with DFS of the patients (HR 3.02; 95% CI 1.66–5.49; p = 0.001) (Fig 1). After adjusting for multiple comparisons and clinical risk factors, the statistically significant association for rs8086 polymorphism remained significant (p<0.05). The adjusted HR for the association between rs8086 genotype (for the recessive model of inheritance) and DFS was 3.08 (95% CI 1.69–5.63, p = 0.046) (Table 3), indicating that patients carrying the *ACSL1* rs8086 T/T genotype had significantly decreased DFS compared with patients carrying the C/T + C/C genotype, with 3-fold higher risk of relapse.

Thus, 3-year DFS in patients carrying the “risk genotype” for rs8086 SNP (T/T) was 54.5% (95%CI: 0.372–0.799) compared with 78.5% (0.736–0.837) in patients with C/T + C/C genotype (in the recessive model of inheritance).

Additionally, genotype for rs8086 polymorphism was analyzed in 40 samples of healthy colon tissues that were obtained from apparently healthy tissues adjacent to tumors from these patients. 40 out of the 40 healthy tissue samples analyzed (100%) had the same genotype for rs8086 that its respective CC sample.

Finally, in the same manner as rs522951, we investigated whether rs8086 polymorphism correlated with gene expression level of *ACSL1*. Genotype-gene expression association study was carried out in a subsample of 210 stage II/III CC patients (141 stage II and 69 stage III CC patients) where gene expression levels of *ACSL1* had been previously determined[11]. The rs8086 SNP showed different *ACSL1* gene expression level according to the genotype in each and every model of inheritance (Table 4; Fig 2). In the additive model of inheritance for rs8086, patients carrying C/C genotype had lower *ACSL1* mRNA levels (mean = 2.37; SD = 3.22) compared with C/T (mean = 3.73; SD = 6.9) and T/T genotype (mean = 5.34; SD = 5.43) with statistically significant differences (p-value KW test = 0.047; p-value ANOVA = 0.019). In the dominant model, patients with C/C genotype had lower *ACSL1* mRNA levels (mean = 2.37; SD = 3.22) than those with C/T + T/T genotype (mean = 3.96; SD = 6.71) with statistically significant differences (p-value KW test = 0.03; p-value ANOVA = 0.039). Finally, in the recessive model the C/C + C/T genotype (mean = 3.09; SD = 5.53) presented the lowest *ACSL1* mRNA levels compared with T/T genotype (mean = 5.34; SD = 5.43) with a trend (p-value KW test = 0.091; p-value ANOVA = 0.109).

## Discussion

Several lines of evidence indicate that LM plays a crucial role in carcinogenesis [11, 12, 17]. Accordingly, expression levels of several related genes have been found to display prognostic and predictive value in CC patients [11, 12, 18].

In order to analyze whether genetic variants in these genes might be mediating this clinical association, we tested whether 57 tagging polymorphisms (including functional variations) located in 7 different LM-related genes previously associated with prognosis predict DFS in 284 patients with stage II/III CC. In addition, we evaluated whether different genotypes of the functional polymorphisms correlated with levels of mRNA expression of the respective genes.

With respect to the analysis of the influence of the 57 analyzed SNPs on the DFS, the results showed a robust association for rs8086 and rs522951 (Table 3; Fig 1) between DFS of the patients and genotype for these polymorphisms within *ACSL1* and *SCD* respectively, genes that codify for two enzymes that have been recently described as a LM driving-force in colon cancer progression [10].

In the multivariate model (adjusting for clinical risk factors and multiple comparisons), only rs8086 confirmed the statistically significant association between genotype (for the recessive model of inheritance) and DFS (Table 3), indicating that patients carrying the *ACSL1* rs8086 T/T genotype had significantly decreased DFS compared with patients carrying the C/T + C/C genotype, with 3-fold higher risk of relapse. Afterwards, in order to assess whether rs8086 and rs522951 were a tumor-specific polymorphisms or by contrast a germline polymorphisms in these CC patients, we analyzed the genotype for both polymorphisms in 40 samples of healthy colon tissues adjacent to tumors. The results showed that 100% of the samples had the same genotype in both, healthy and tumor samples for rs8086 and rs522951, indicating that rs8086 as well rs522951 are germline polymorphisms not specific of the tumor. These data is of special relevance because if these results are further confirmed in validation cohorts, rs8086 could be used as non-invasive biomarker of prognosis in stage II/III CC patients, whose genomic DNA could be extracted from buccal epithelial cells in a non-invasive and single manner. In this sense, further subgroup analysis was performed to confirm whether both stages showed similar performance. As it is shown in S1 Fig, despite reducing the number of samples included in the stratified analysis, in both stages these genetic variants were associated with worse clinical outcome, with increased significance in stage II accordingly with previous studies of the group (10–11). In addition, this analysis showed also association between these genetic variants and worse clinical outcome in patients treated with chemotherapy and a trend in those without treatment (S2 Fig), in which the number of patients is low as it usually found in clinical practice, contributing in part to the lack of statistical significance in this last case.

In addition, since rs8086 was a functional polymorphism located in the 3’-UTR region of *ACSL1* gene, we aim to evaluate whether rs8086 genotype could be correlated with *ACSL1* mRNA level and influence the prognosis of these CC patients according to the data previously obtained [11]. Hence, genotype-gene expression level association in a subsample of 210 stage II/III CC patients was carried out and the results showed that rs8086 exhibited different *ACSL1* gene expression level depending on rs8086 genotype for all model of inheritance (Table 4; Fig 2), highlighting that the decrease of the *ACSL1* mRNA levels were directly proportional to the number of C allele.

In summary, in this study T/T genotype for rs8086 is associated with worse clinical outcome acting as a “risk genotype” in these CC patients and simultaneously correlates with high *ACSL1* mRNA levels, which in turn had previously been associated also with worse clinical outcome in these patients [11] probably mediated by the induction of an invasive phenotype [10].

To date, 50 SNPs located in 40 loci have been associated with the risk of CRC by genome-wide association studies (GWASs) [2] and recent evidences indicate a potential prognostic and predictive value in CC for polymorphisms in genes involved in a variety of cellular process such as cell cycle control [19], inflammation [2, 20, 21], Hedgehog signaling pathway [14], tight junction [22], DNA repair or drug metabolism and drug resistance [23–26]. In the context of LM, a polymorphism in *ApoE* gene has been associated with CRC risk and prognosis in a gender-dependent manner [27]. However, to our knowledge, this is the first report suggesting a relationship between *ACSL1* polymorphism and clinical outcome in stage II/III patients with CC.

*ACSL1* is an isozyme of Acyl-CoA synthetase (ACSL) family, which catalyzes the conversion of long chain fatty acids to acyl-CoA, which is critical for phospholipid and triglyceride synthesis, lipid modification of proteins as well as for fatty acid β-oxidation. Due to its relevant function in metabolic regulation, it has been recently shown to display an important role in cancer cell survival, apoptosis inhibition and epithelial-mesenchymal transition [10, 11, 28]. Given the role of *ACSL1* in carcinogenesis and the influence of genetic polymorphisms in regulation of gene expression and function, it is inferred that polymorphisms in this gene might exert an influence on cancer susceptibility and progression. Furthermore, we have recently reported that this enzyme constitutes a promising therapeutic target for CC therapy [10]. These findings suggest that rs8086 *ACSL1* polymorphism may serve as a useful prognostic biomarker, but due to the strong evidence about the biological significance of this gene and the rather limited number of cases in our study, further independent studies are needed to evaluate the significance of our findings in the clinic. Moreover, and related to the potential clinical application of this polymorphism as a non-invasive biomarker, an extensive and comparative genotyping analysis for rs8086 of genomic DNA extracted from buccal epithelial cells (in saliva) or blood cells (in plasma) compared to FFPE tumor samples from stage II and III CC patients are still needed.

In conclusion, our study identified a genetic variant in the 3’-UTR region of *ACSL1* gene (rs8086) that may play a significant role in predicting outcomes of stage II/III patients with CC, so that patients with T/T genotype had a significantly higher risk of tumor recurrence than those carrying at least one C allele. The molecular mechanisms by which rs8086 *ACSL1* polymorphism affects tumor behavior and recurrence are under investigation. Since rs8086 *ACSL1* polymorphism is located at 3´-UTR region, and the SNP functional prediction tool (F-SNP) has shown that a single nucleotide change from C to T may alter miRNAs binding sites in this gene, modulation of transcription has been suggested. Consistent with this suggestion, additional studies are needed to better elucidate the mechanisms underlying these putative associations. Furthermore, taking into consideration the reported cooperative network of *ACSL1*, *ACSL4* and *SCD* and its role in the progression of colorectal cancer, the future direction of the current research will be to test the mutation status of all three genes together and examine the putative association with the expression level of these genes and with clinical outcome in stage II/III CC patients.

## Supporting Information

S1 Fig Kaplan-Meier curve of *ACSL1* SNP rs8086 and *SCD* SNP rs522951 on DFS stratified by stage (stage II vs III). P-value was calculated by Log-rank test.
(TIF)

S2 Fig Kaplan-Meier curve of *ACSL1* SNP rs8086 and *SCD* SNP rs522951 on DFS stratified by chemotherapy (patients with chemotherapy vs patients without chemotherapy). P-value was calculated by Log-rank test.
(TIF)

S1 Table Univariate cox regression analysis for Disease-free survival of the clinical variables in stage II and III CC patients. HR (95% CI), hazard ratio and corresponding 95% confidence interval from univariate cox proportional hazards analysis; P, p value from univariate cox regression analysis.
(XLSX)

S2 Table Genotype frequency for rs8086 (*ACSL1*) and rs522951 (*SCD*) SNPs in stage II and III CC patients. N, number of cases in each genotype; %, percentage of cases in each genotype; ND, Not determined.
(XLSX)
