# Supplementary figures and images for: 3’UTR Polymorphism in ACSL1 Gene Correlates with Expression Levels and Poor Clinical Outcome in Colon Cancer Patients
Source: PLoS One. 2016 Dec 19;11(12):e0168423. doi: 10.1371/journal.pone.0168423 (PMC5167383; doi:10.1371/journal.pone.0168423)

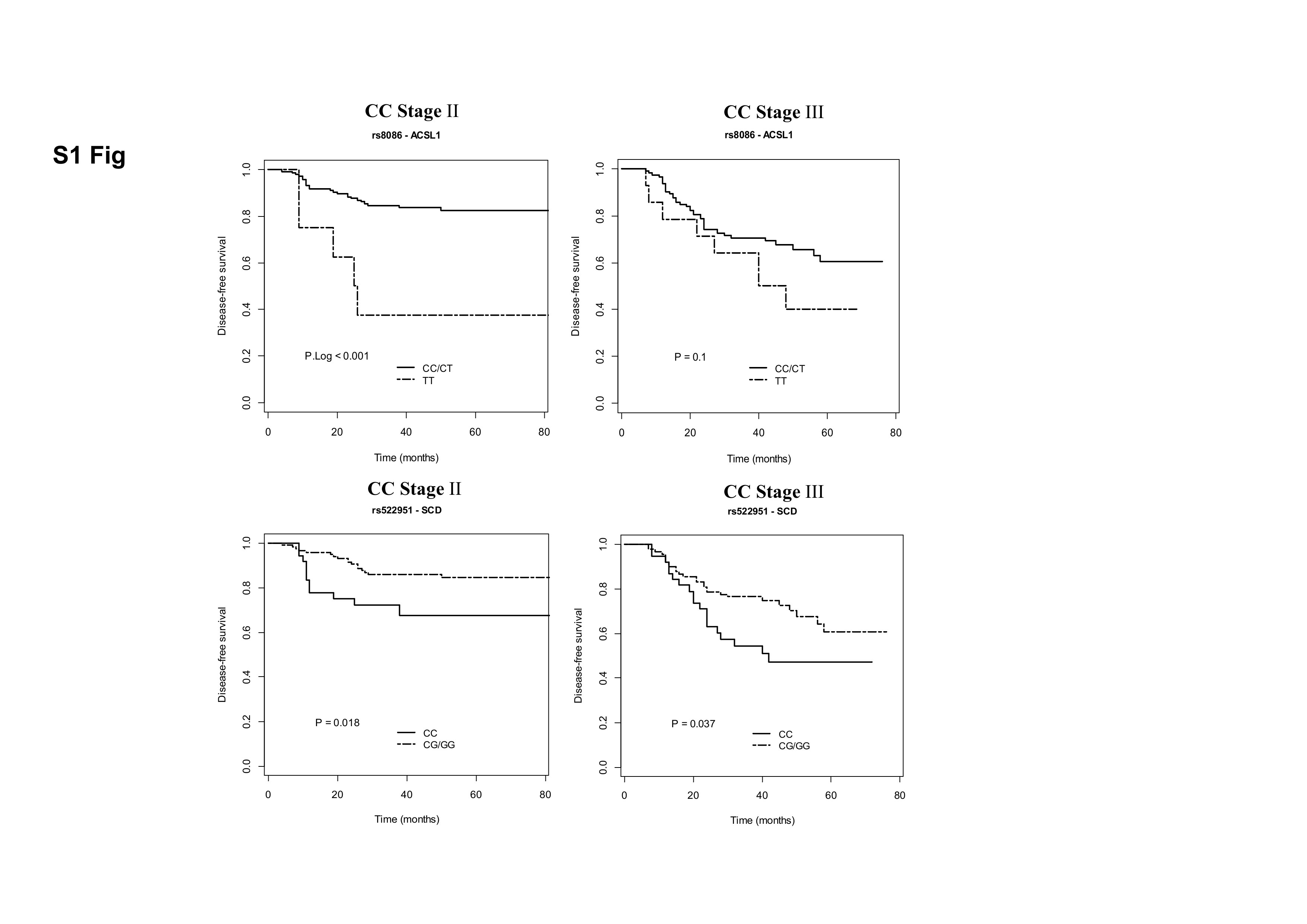

Supplement: S1 Fig — P-value was calculated by Log-rank test. (TIF) [file pone.0168423.s001.tif]

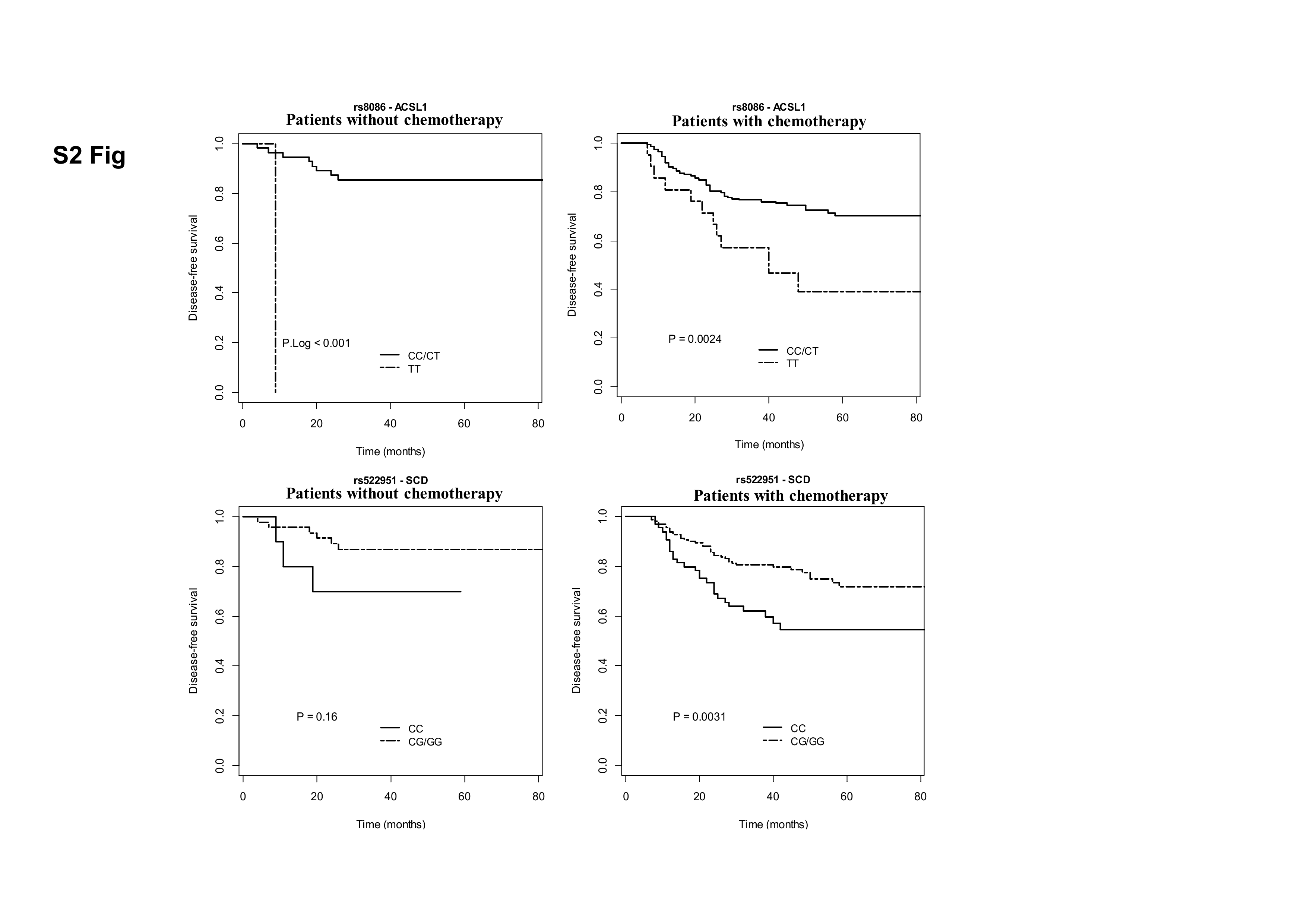

Supplement: S2 Fig — P-value was calculated by Log-rank test. (TIF) [file pone.0168423.s002.tif]
